# Ventricular and Atrial Pressure—Volume Loops: Analysis of the Effects Induced by Right Centrifugal Pump Assistance

**DOI:** 10.3390/bioengineering9050181

**Published:** 2022-04-20

**Authors:** Beatrice De Lazzari, Attilio Iacovoni, Massimo Capoccia, Silvia Papa, Roberto Badagliacca, Domenico Filomena, Claudio De Lazzari

**Affiliations:** 1 Department of Human Movement and Sport Sciences, “Foro Italico” 4th University of Rome, 00135 Rome, Italy; b.delazzari@studenti.uniroma4.it; 2 Department of Cardiology, ASST-Papa Giovanni XIII Hospital, 24127 Bergamo, Italy; aiacovoni@asst-pg23.it; 3 Department of Cardiac Surgery, Leeds General Infirmary, Leeds Teaching Hospitals NHS Trust, Leeds LS1 3EX, UK; 4 Department of Biomedical Engineering, University of Strathclyde, Glasgow G4 0NW, UK; 5 Department of Clinical, Internal Anesthesiology and Cardiovascular Sciences, “Sapienza” University of Rome, 00185 Rome, Italy; silvia.papa@uniroma1.it (S.P.); roberto.badagliacca@uniroma1.it (R.B.); domenico.filomena@uniroma1.it (D.F.); 6 National Research Council, Institute of Clinical Physiology (IFC-CNR), 00185 Rome, Italy; claudio.delazzari@ifc.cnr.it; 7 Faculty of Medicine, Teaching University Geomedi, Tbilisi 0114, Georgia

**Keywords:** heart failure, RVAD, pressure-volume loop, lumped parameter model, software simulation, cardiovascular modelling, rotational pump speed, right-left heart interaction

## Abstract

The main indications for right ventricular assist device (RVAD) support are right heart failure after implantation of a left ventricular assist device (LVAD) or early graft failure following heart transplantation. We sought to study the effects induced by different RVAD connections when right ventricular elastance (Ees_RIGHT_) was modified using numerical simulations based on atrial and ventricular pressure–volume analysis. We considered the effects induced by continuous-flow RVAD support on left/right ventricular/atrial loops when Ees_RIGHT_ changed from 0.3 to 0.8 mmHg/mL during in-series or parallel pump connection. Pump rotational speed was also addressed. Parallel RVAD support at 4000 rpm with Ees_RIGHT_ = 0.3 mmHg/mL generated percentage changes up to 60% for left ventricular pressure–volume area and external work; up to 20% for left ventricular ESV and up to 25% for left ventricular EDV; up to 50% change in left atrial pressure-volume area (PVLA_L-A_) and only a 3% change in right atrial pressure–volume area (PVLA_R-A_). Percentage variation was lower when Ees_RIGHT_ = 0.8 mmHg/mL. Early recognition of right ventricular failure followed by aggressive treatment is desirable, so as to achieve a more favourable outcome. RVAD support remains an option for advanced right ventricular failure, although the onset of major adverse events may preclude its use.

## 1. Introduction

Acute right ventricular (RV) failure may develop in the context of acute decompensated heart failure, acute myocardial infarction, pulmonary embolism, fulminant myocarditis, decompensated pulmonary hypertension, post-cardiotomy shock, orthotopic heart transplant, and often after the insertion of a left ventricular assist device (LVAD) [1]. This may also be the case when a long-term right ventricular assist device (RVAD) is required for end-stage RV failure from combined pre- and post-capillary pulmonary hypertension (PH) [2].

The main indications for RVAD support are right heart failure after LVAD implantation or early graft failure following orthotopic heart transplantation. About 30–40% of patients will need RVAD support after LVAD implantation [3,4,5,6]. Markers of illness severity including evidence of end-organ dysfunction and haemodynamic profile are associated with the need for RVAD support within two weeks following LVAD insertion [7]. The prognostic role of the right ventricle is now being acknowledged in the context of left-sided heart failure [8]. Failure of systolic function adaptation (homeometric adaptation described by Anrep’s law) leads to increased dimensions (heterometric adaptation described by Starling’s law) with a negative effect on diastolic ventricular interactions [9]. RV-PA coupling has significant reserve in the context of elevated RV afterload, although the level of uncoupling that leads to RV failure remains not completely defined [10]. Better understanding of the pathophysiology of right ventricular (RV) failure may well help with its initial medical management and timing of mechanical circulatory support. Prolonged survival by effective medical treatment becomes the grounds for the development of right heart failure secondary to chronic left ventricular dysfunction. Patients remain compensated as long as the right ventricle is functional. The ability to track the RV based on better monitoring of afterload and functional reserve may help change the course of the disease before the RV reaches the threshold that may limit both medical and LVAD treatment [11].

However, the lack of data does not allow for an in-depth analysis of right ventricular and atrial behaviour during RVAD support. An attempt in this direction can be made using a numerical simulator of the cardiovascular system with a view to reproduce pathological conditions requiring right ventricular assistance.

The aim of our study was the trend analysis in terms of percentage variation of the haemodynamic and energetic variables of both ventricles and atria in different cardiovascular conditions reproduced by changing right ventricular elastance from 0.3 to 0.8 mmHg/mL during RVAD support with different rotational speeds. 

The first step of this work required the implementation of two new modules within CARDIOSIM© software simulation platform, which would reproduce the characteristics of the left circulatory network and a continuous flow centrifugal pump (RVAD) to be connected either in series or parallel to the right ventricle. The new modules were based on a 0-D (lumped-parameter) numerical model including input and output cannula of the RVAD.

We simulated RVAD support following in series and parallel connection driven by different rotational speeds in a heart failure setting. Subsequently, the right ventricular elastance was increased from 0.3 to 0.8 mmHg/mL in a stepwise manner. During each setting, we focused our attention on the following hemodynamic and energetic variables:

✓ Right and left ventricular end-systolic volume (ESV_R-V_ and ESV_L-V_);
✓ Right and left ventricular end-diastolic volume (EDV_R-V_ and EDV_L-V_);
✓ Stroke volume (SV);
✓ Right and left ventricular external work (EW_R-V_ and EW_L-V_) and pressure-volume area (PVA_R-V_ and PVA_L-V_);
✓ Right and left atrial end-systolic volume (ESV_R-A_ and ESV_L-A_);
✓ Right and left atrial end-diastolic volume (EDV_R-A_ and EDV_L-A_);
✓ Right and left atrial pressure-volume loop area (PVLA_R-A_ and PVLA_L-A_);
✓ Cardiac output (CO);
✓ Systolic, diastolic, and mean systemic aortic pressure (AoP);
✓ Systolic, diastolic, and mean pulmonary arterial pressure (PAP);
✓ Pulmonary capillary wedge pressure (PCWP);
✓ Right and left atrial pressure (RAP and LAP).

## 2. Materials and Methods

### 2.1. The Heart and Circulatory Numerical Network

**Figure 1:**
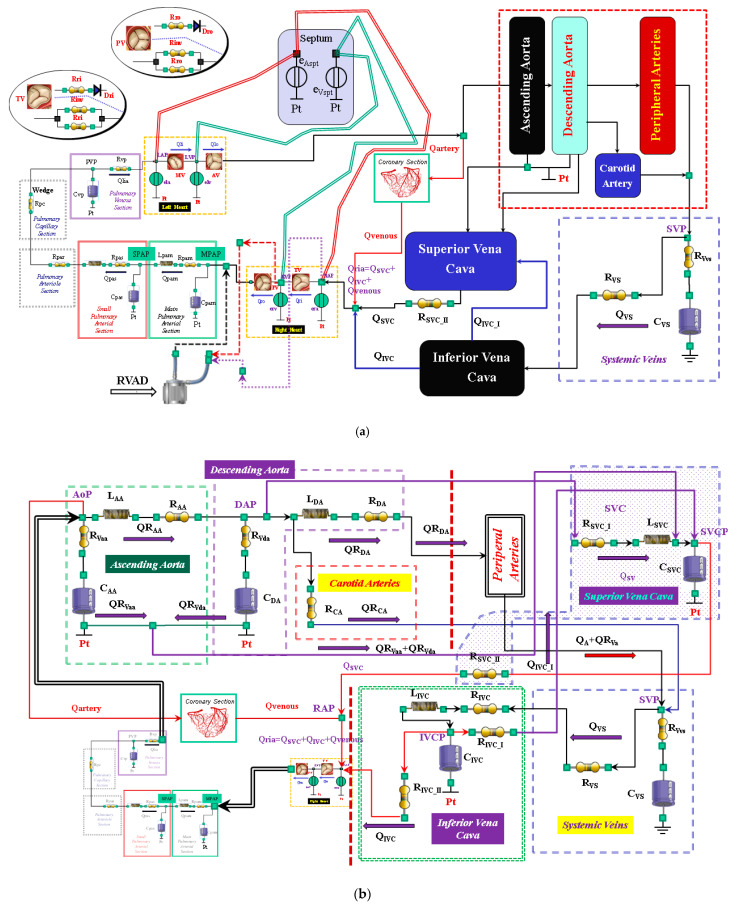

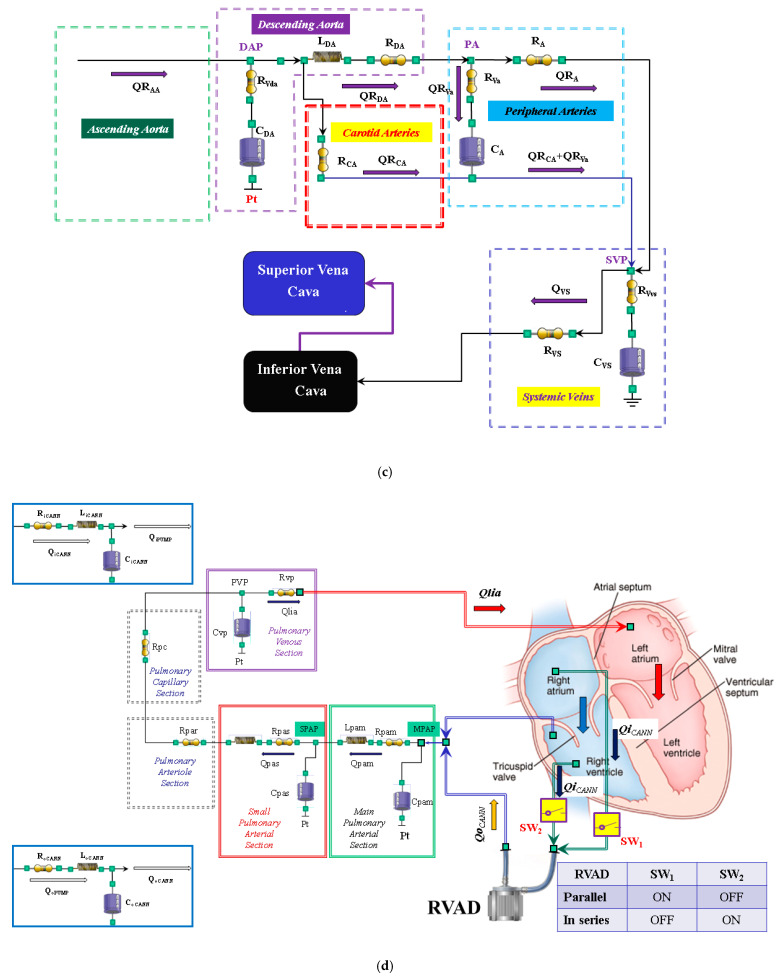
(**a**) Electric analogue of the cardiovascular system. The network is assembled with septum, left and right heart, main and small pulmonary arterial sections, pulmonary arteriole and capillary sections, and the pulmonary venous section. The left circulation includes ascending and descending aorta compartments, peripheral arteries, and carotid artery sections, coronary circulation, superior and inferior vena cava sections, and systemic veins compartment. RVAD is the right ventricular assist device. Table 2 lists the symbols used. (**b**) The behaviour of the ascending aorta is simulated with resistances R_AA_ and R_Vaa_, inertance L_AA_ and compliance C_AA_. QR_AA_ is the flow through the resistance and inertance. The descending aorta is implemented with resistances R_DA_ and R_Vda_, inertance L_DA_ and compliance C_DA_. QR_DA_ is the flow through the resistance (R_DA_) and inertance (L_DA_). The carotid arteries section is reproduced with a simple resistance (R_CA_). The superior vena cava module consists of resistances R_SVC_I_ and R_SVC_II_, inertance L_SCV_, and compliance C_SVC_. The inferior vena cava module is modelled with resistances R_IVC_, R_IVC_I_, and R_IVC_II_; inertance L_IVC_; and compliance C_IVC_. The intrathoracic pressure (Pt) affects compliances C_AA_, C_DA_, C_IVC_, and C_SVC_. Table 2 lists the symbols used. (**c**) The peripheral arteries module is modelled with resistances R_A_ and R_Va_ and compliance C_A_. The resistor R_Va_ accounts for viscous losses of the vessels wall. QR_A_ is the blood flow outside the compartment; it is a part of the blood that reaches the systemic veins compartment. (**d**) Schematic representation of RVAD connection. When the right ventricular assist device is connected in parallel, blood is removed from the right atrium (SW_1_ = ON and SW_2_ = OFF) and ejected into the pulmonary artery. When RVAD is connected in series, blood is removed from the right ventricle (SW_1_ = OFF and SW_2_ = ON) and ejected into the pulmonary artery. The input (output) RVAD cannula is modelled with RLC elements. Q_oPUMP_ (Q_iPUMP_) is the output (inlet) flow rate from the pump. Q_oCANN_ (Q_iCANN_) is the output (inlet) flow rate from the cannula. The electrical analogue of the pulmonary circulation is described in [22] (Reprinted with permission from Ref. [22], Copywright© 1991–2019 C. De Lazzari).

CARDIOSIM© software (Rome, Italy) platform has been previously described [12,13,14,15,16,17]. The modules of the software simulator are: systemic and pulmonary arterial section, systemic and pulmonary venous section, and coronary circulation. Native left and right ventricles, atria, and septum reproduce the entire cardiac activity; they are implemented in a single module (Figure 1) using the time-varying elastance concept [13,14]. The ventricular, atrial, and septal activity is synchronized with the electrocardiographic (ECG) signal [14]. Using the modified time-varying elastance theory, inter-ventricular septum (IVS) interaction and instantaneous left and right ventricular pressure can be reproduced by [14,18]:

(1) $$\left\{ \begin{array}{l} P_{lv}(t)=\left[\frac{e_{Vsp}(t)\cdot e_{lv}(t)}{e_{lv}(t)+e_{Vsp}(t)}\right]\cdot \left[V_{lv}(t)-V_{lv,0}\right]+\left[\frac{e_{lv}(t)}{e_{lv}(t)+e_{Vsp}(t)}\right]\cdot P_{rv}(t)+\left[\frac{e_{Vsp}(t)}{e_{lv}(t)+e_{Vsp}(t)}\right]\cdot P_{lv,0}\\ P_{rv}(t)=\left[\frac{e_{Vsp}(t)\cdot e_{rv}(t)}{e_{Vsp}(t)+e_{rv}(t)}\right]\cdot \left[V_{rv}(t)-V_{rv,0}\right]+\left[\frac{e_{rv}(t)}{e_{Vsp}(t)+e_{rv}(t)}\right]\cdot P_{lv}(t)+\left[\frac{e_{Vsp}(t)}{e_{Vsp}(t)+e_{rv}(t)}\right]\cdot P_{rv,0} \end{array}\right.$$

In the same way, the inter-atrial septum (IAS) interaction and the left and right instantaneous atrial pressure can be reproduced by:

(2) $$\left\{ \begin{array}{l} P_{la}(t)=\left[\frac{e_{Asp}(t)\cdot e_{la}(t)}{e_{la}(t)+e_{Asp}(t)}\right]\cdot \left[V_{la}(t)-V_{la,0}\right]+\left[\frac{e_{la}(t)}{e_{la}(t)+e_{Asp}(t)}\right]\cdot P_{ra}(t)+\left[\frac{e_{Asp}(t)}{e_{la}(t)+e_{Asp}(t)}\right]\cdot P_{la,0}\\ P_{ra}(t)=\left[\frac{e_{Asp}(t)\cdot e_{ra}(t)}{e_{Asp}(t)+e_{ra}(t)}\right]\cdot \left[V_{ra}(t)-V_{ra,0}\right]+\left[\frac{e_{ra}(t)}{e_{Asp}(t)+e_{ra}(t)}\right]\cdot P_{la}(t)+\left[\frac{e_{Asp}(t)}{e_{Asp}(t)+e_{ra}(t)}\right]\cdot P_{ra,0} \end{array}\right.$$

The symbols used in Equations (1) and (2) have been listed in Table 1. These features allow the simulation of inter-ventricular and intra-ventricular dyssynchrony [19]. 

Specific modules of the coronary circulation (Figure 1) are also available on the CARDIOSIM© platform [12]. 

For the purposes of our study, we assembled the cardiovascular network with the new module of the systemic circulation, whilst the behaviour of the heart is modelled as described in [12,13]. The systemic venous section [12,15] and the entire pulmonary circulation [16,17,20,21] are modelled as described in the current literature. We have selected the module presented in [22] for the coronary circulation. The tricuspid, mitral, pulmonary, and aortic valves are modelled with an ideal diode: when the pressure across the valve is positive, the valve opens and allows the flow of blood; when the pressure is less than or equal to zero, the valve closes and the flow of blood is zero [12,13,14,23].

### 2.2. New Lumped-Parameter Model of the Systemic Circulation

The new module is described in Figure 1 using resistance, inertance, and compliance (RLC) elements. The systemic network consists of the following compartments: ascending and descending aorta, carotid artery, peripheral arteries, and superior and inferior vena cava (Figure 1a). 

The ascending (descending) aorta is modelled using two resistances R_AA_ and R_Vaa_ (R_DA_ and R_Vda_), inertance L_AA_ (L_DA_), and compliance C_AA_ (C_DA_). A single resistance (R_CA_) reproduces the behaviour of the carotid district (Figure 1b). The peripheral arterial circulation is reproduced with resistances R_A_ and R_Va_ and with compliance C_A_ (Figure 1c). The superior vena cava compartment consists of resistances R_SVC_I_ and R_SVC_II_, inertance L_SVC_, and compliance C_SVC_. The systemic venous network is implemented using compliance C_VS_ and resistances R_VS_ and R_Vvs_ (Figure 1c). Finally, the inferior vena cava district is modelled with resistances R_IVC_, R_IVC_I_, and R_IVC_II_; inertance L_IVC_; and compliance C_IVC_ (Figure 1b). The resistances R_Vaa_, R_Vda_, R_Va_, and R_Vvs_ account for viscous losses of the vessel wall. Pt is the mean intrathoracic pressure. All the symbols of the cardiovascular network are listed in Table 2.

### 2.3. Right Ventricular Assist Device (RVAD)

A 0-D numerical model described in [16] was used to implement a centrifugal pump that reproduced the behaviour of the right ventricular assist device. The pump can be connected in series or parallel to the right ventricle with two cannulae modelled using RLC elements (Figure 1d). When the RVAD removes blood from the right atrium (parallel connection—SW1 = ON and SW2 = OFF in Figure 1d), the flow through the inlet cannula is:

(3) $$\left(RAP-\Delta P\right)=Qi_{CANN}\cdot Ri_{CANN}+\left(\frac{d}{dt}Qi_{CANN}\right)\cdot Li_{CANN}$$

(4) $$\left(\frac{d}{dt}\Delta P\right)\cdot Ci_{CANN}=Q_{PUMP}-Qi_{CANN}$$

*RAP* is the right atrial pressure, *Li_CANN,_ Ri_CANN_*, and *Ci_CANN_* are the inertance, resistance, and compliance of the inlet cannula, respectively (Figure 1d). *Qi_CANN_* (*Q_PUMP_*) is the flow through the inlet cannula (generated by the centrifugal pump). Δ*P* is the pressure on the pump head.

When the RVAD removes blood from the right ventricle (in series connection—SW1 = OFF and SW2 = ON in Figure 1d), Equation (3) becomes:

(5) $$\left(RVP-\Delta P\right)=Qi_{CANN}\cdot Ri_{CANN}+\left(\frac{d}{dt}Qi_{CANN}\right)\cdot Li_{CANN}$$

*RVP* is the right ventricular pressure.

When the RVAD ejects blood, the flow through the outlet cannula is:

(6) $$\left(\Delta P-MPAP\right)=Qo_{CANN}\cdot Ro_{CANN}+\left(\frac{d}{dt}Qo_{CANN}\right)\cdot Lo_{CANN}$$

(7) $$\left(\frac{d}{dt}\Delta P\right)\cdot Co_{CANN}=Qo_{PUMP}-Qo_{CANN}$$

*MPAP* is the mean pulmonary artery pressure (Figure 1d), *Ro_CANN_* and *Co_CANN_* are the inertance, resistance, and compliance of the outlet cannula, respectively. *Qo_CANN_* is the flow through the outlet cannula. 

### 2.4. Simulation Protocol

For the purposes of our simulations, we considered the values for right and left ventricular elastance that could reproduce a realistic diseased heart. According to the available literature, normal values for right ventricular elastance fluctuate around 1 mmHg/mL [24,25] and range from 1.6 to 5 mmHg/mL for left ventricular elastance [17,26]. Therefore, we considered Ees_LEFT_ = 0.7 mmHg/mL for the left ventricle and Ees_RIGHT_ = 0.3 mmHg/mL for the right ventricle as reference values for a failing heart. Our simulation approach consisted of three steps. In the first step after setting HR = 90 bpm, the slope of left ventricular End-Systolic Pressure-Volume Relationship (ESPVR) Ees_LEFT_ = 0.7 mmHg/mL, and the slope of right ESPVR Ees_RIGHT_ = 0.3 mmHg/mL [27], the simulator generated the following values: cardiac output (CO) 4.51 L/min, aortic systolic (diastolic) pressure 82.1 (60.4) mmHg, mean right atrial pressure 23.3 mmHg, pulmonary systolic (diastolic) pressure 51.2 (31.6) mmHg, and pulmonary capillary wedge pressure 20.7 mmHg. The mean pressure (flow) value was calculated as the mean value of all blood pressure (flow) measurements during a cardiac cycle.

In the second step, the slope of right ESPVR Ees_RIGHT_ = 0.3 mmHg/mL was set to 0.4, 0.5, 0.6, 0.7, and 0.8 mmHg/mL [10,28] and for each value the parameters described above were measured.

In the third step, RVAD support was applied both in series and parallel mode with rotational speed of 2000, 2500, 3000, 3500, and 4000 rpm. In the fourth step, the slope of the right ESPVR was changed from 0.3 to 0.8 mmHg/mL (0.1 mmHg/mL stepwise increase) during RVAD support connected in series and parallel mode. The measured parameters were those described above.

Considering that we did not measure data in patients undergoing RVAD support to allow us to reproduce their haemodynamic conditions with our numerical simulator, and given that literature data are largely incomplete, we decided against a direct data comparison. Therefore, we considered percentage variation to evaluate the trend of the effects induced by RVAD support on the haemodynamic and energetic variables in line with other available simulation work.

## 3. Results

**Figure 2:**
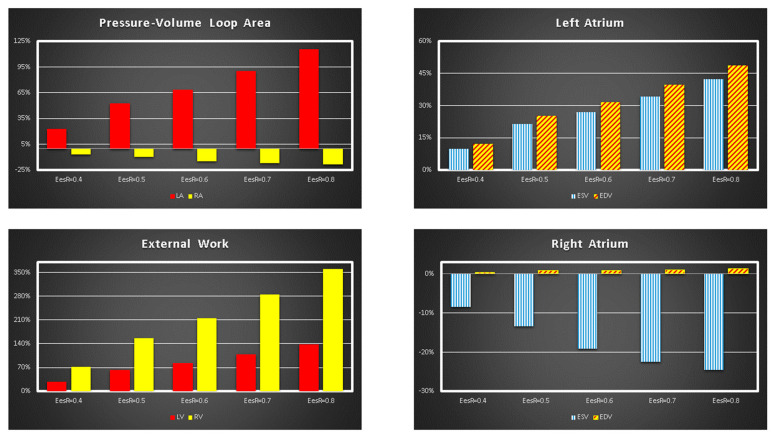
Relative changes calculated in comparison to Ees_RIGHT_ = 0.3 mmHg/mL for different Ees_RIGHT_ values (0.4–0.8 mmHg/mL). The relative changes of the pressure–volume loop area of the left and right atrium (external work of the left and right ventricle) are reported in the left upper (lower) panel. The right upper (lower) panel shows the relative changes of left (right) atrial end systolic and end diastolic volume.

**Figure 3:**
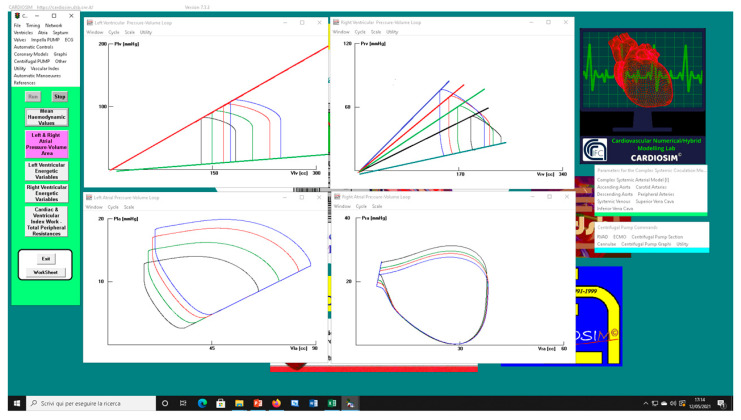
Screen output from CARDIOSIM© software simulator. The left (right) upper panel shows four left (right) ventricular pressure–volume loops obtained by setting EesR ≡ Ees_RIGHT_ = 0.3 mmHg/mL (black line), EesR ≡ Ees_RIGHT_ = 0.4 mmHg/mL (green line), EesR ≡ Ees_RIGHT_ = 0.5 mmHg/mL (red line), and EesR ≡ Ees_RIGHT_ = 0.6 mmHg/mL (blue line), respectively. The left (right) lower panel shows four left (right) atrial pressure–volume loops obtained by changing the slope of the right ventricular elastance as previously described.

**Figure 4:**
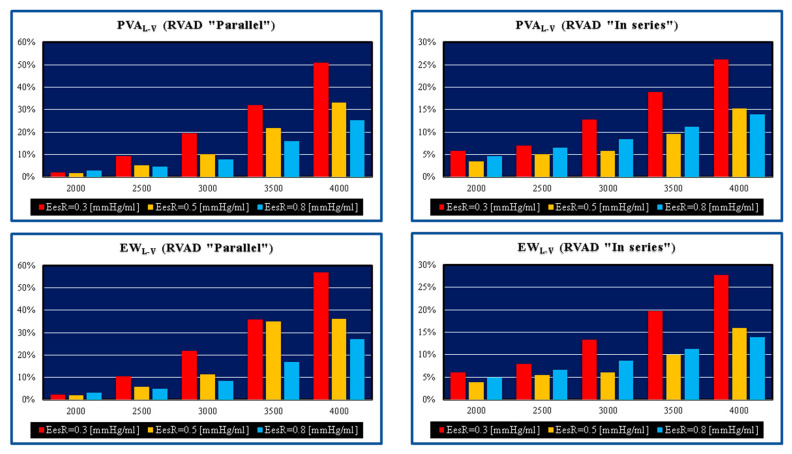
Relative changes calculated in comparison to pathological conditions (EesR ≡ Ees_RIGHT_ = 0.3, 0.5, and 0.8 mmHg/mL) for different types of RVAD connection and different rotational speeds. For each values of Ees_RIGHT_, the relative change was calculated when the RVAD was connected in parallel and in series. The left upper (lower) panel shows the relative changes in the left ventricular pressure–volume area (external work) when the RVAD was connected in parallel to the right ventricle. The right upper (lower) panel shows the relative changes in the left ventricular pressure–volume area (external work) when the RVAD was connected in series to the right ventricle.

**Figure 5:**
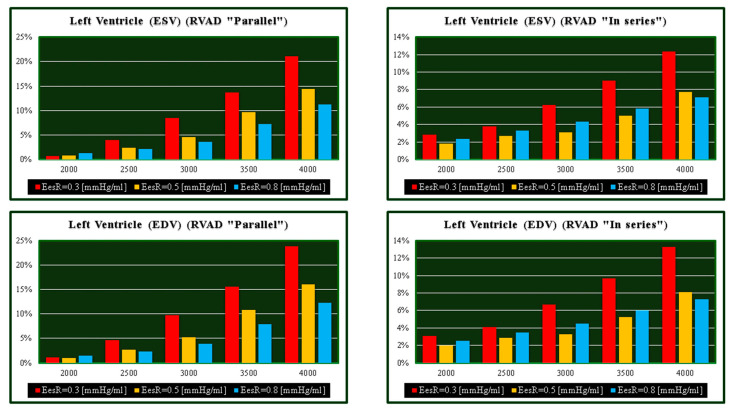
Relative changes calculated in comparison to pathological conditions (EesR ≡ Ees_RIGHT_ = 0.3, 0.5, and 0.8 mmHg/mL) for different types of RVAD connection and different rotational speeds. For each value of Ees_RIGHT_, the relative change was calculated when the RVAD was connected in parallel and “in series” mode. The left upper (lower) panel shows the relative changes in the left ventricular end systolic (end diastolic) volume when the RVAD was connected in parallel to the right ventricle. The right upper (lower) panel shows the relative changes in the left ventricular end systolic (end diastolic) volume when the RVAD was connected in series to the right ventricle.

**Figure 6:**
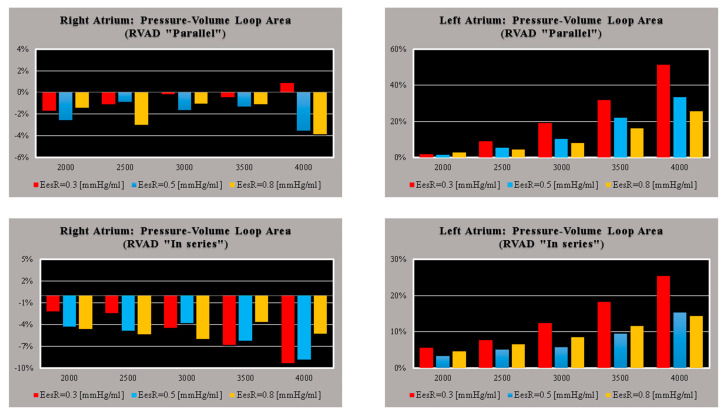
Relative changes calculated in comparison to pathological conditions (EesR ≡ Ees_RIGHT_ = 0.3, 0.5, and 0.8 mmHg/mL) for different types of RVAD connection and different rotational speeds. For each value of Ees_RIGHT_, the relative change was calculated when the RVAD was connected in parallel and in-series mode. The left (right) upper panel shows the relative changes in the right (left) atrium pressure–volume loop area when the RVAD was connected in parallel to the right ventricle. The left (right) lower panel shows the relative changes in the right (left) atrium pressure–volume loop area when the RVAD was connected in series to the right ventricle.

Figure 2 shows the effects induced by different values for ESPVR slope (Ees_RIGHT_) on left and right atrial pressure–volume loop area (left upper panel). The percentage changes calculated with respect to the reference value for Ees_RIGHT_ = 0.3 mmHg/mL have been listed. The red bars show the percentage change with respect to the reference value for PVLA_L-A_ increase at high Ees_RIGHT_ values. On the contrary, the PVLA_R-A_ (yellow bars) decreases when the percentage change is referred to high Ees_RIGHT_ values. These results show that when Ees_RIGHT_ increases from 0.3 to 08 mmHg/mL, the area of the pressure–volume loop of the right atrium decreases, whilst the area of the pressure–volume loop of the left atrium increases (Figure 3). The left and right lower panel shows the left and right atrial pressure–volume loop obtained with different Ees_RIGHT_ values. The black pressure–volume loop was obtained by setting Ees_RIGHT_ = 0.3 mmHg/mL, while the blue pressure–volume loop was obtained by setting Ees_RIGHT_ = 0.6 mmHg/mL. The left (left side) and the right (right side) ventricular pressure–volume loops are placed in the upper panels. When Ees_RIGHT_ increases, EW_L_V_ and PVA_L-V_ (left upper panel) increase leading to right-sided shift with increased left ESV and EDV. The effect induced by different Ees_RIGHT_ values on left and right ventricular EW (EW_L-V_ and EW_R-V_) are reported in Figure 2 (left lower panel). The effect induced on EDV and ESV of the left (right) atrium is reported in the upper (lower) right panel (Figure 2). The effect induced on the right atrial ESV is more evident than the one produced on right atrial EDV. Figure 3 (right upper panel) shows that an increase in Ees_RIGHT_ leads to an increase in right ventricular external work and PVA_R-V_ with left-sided shift and a decrease of both right ESV and EDV. Figure 4 shows the effect induced by RVAD support driven in parallel connection (left panels) on left ventricular PVA_L-V_ (left upper panel) and EW_L-V_ (left lower panel). The effect induced on PVA_L-V_ and EW_L-V_ when the RVAD is driven in series is available in the right panels. The data were measured for different values of Ees_RIGHT_ (0.3, 0.5, and 0.8 mmHg/mL) and with increasing RVAD rotational speed (2000, 2500, 3000, 3500, and 4000 rpm). For each rotational speed and Ees_RIGHT_ values, the percentage changes calculated with respect to the reference value measured in pathological conditions have been listed (Figure 4). The highest percentage changes in PVA_L-V_ and EW_L-V_ (50% or more for both variables) were recorded in a diseased condition with right ventricular elastance set to Ees_RIGHT_ = 0.3 mmHg/mL during in-parallel RVAD assistance with pump rotational speed at 4000 rpm. Figure 5 shows the effect induced by in-parallel RVAD support on left ventricular ESV_L-V_ (left upper panel) and EDV_L-V_ (left lower panel). The effect induced on ESV_L-V_ (right upper panel) and EDV_L-V_ (right lower panel) by in-series RVAD support is also available for comparison purposes. When the rotational speed of the pump was set to 2000 rpm, the percentage changes for each value of right ventricular ESPVR slope were no more than 3%. The effects induced by a different rotational speed of the RVAD on left and right atrial pressure–volume loop area are available in Figure 6 for three different values of right ESPVR slope. In addition, for each value of the slope, the effect induced by the pump has been calculated in percentage terms with respect to the value obtained without assistance. In the case of the right atrium, we observed a higher percentage reduction of the loop area (from 2 to 9%) during in-series assistance (left lower panel). In the case of parallel connection (left upper panel), the percentage reduction of the pressure–volume loop area of the right atrium becomes more evident at high values for both the slope and the pump rotational speed. Right heart assistance produces significant effects on the left atrium loop area (right panels). Percentage variations between 20 and 50% are observed when the RVAD is connected in parallel to the right ventricle (right upper panel). Whether in-series or in-parallel assistance is considered, the most significant percentage variations are observed when the right ventricular ESPVR slope is set to Ees_RIGHT_ = 0.3 mmHg/mL.

**Figure 7:**
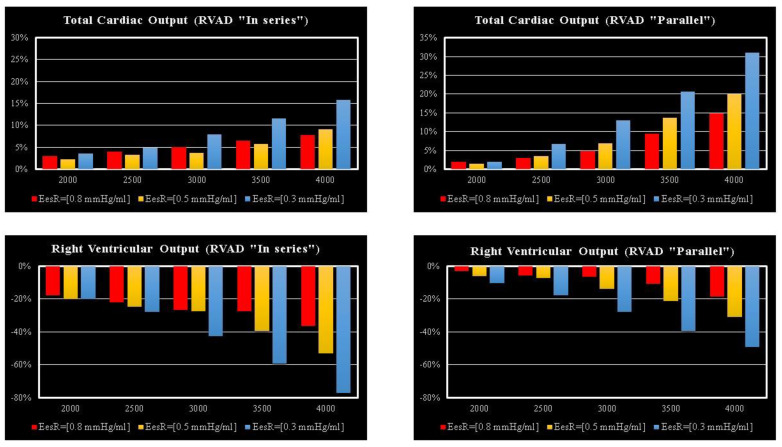
Relative changes calculated in comparison to pathological conditions (EesR ≡ Ees_RIGHT_ = 0.3, 0.5, and 0.8 mmHg/mL) for different types of RVAD connection and different rotational speeds. For each value of Ees_RIGHT_, the relative change was calculated when the RVAD was connected in parallel and in-series mode. The left (right) upper panel shows the relative changes in the total cardiac output (right ventricular flow output plus RVAD flow output) when the RVAD was connected in series (parallel) to the right ventricle. The left (right) lower panel shows the relative changes in the right ventricular flow output when the RVAD was connected in series (parallel) to the right ventricle.

The effects induced by RVAD support on the right ventricular flow output and on the total cardiac output are shown in Figure 7. RVAD parallel assistance generated a lower reduction in right ventricular output compared to in-series assistance for each Ees_RIGHT_ value and each pump’s rotational speed. More specifically, when the pump speed was set to 4000 rpm, parallel assistance induced a reduction between 20 and 45% compared to the baseline value, while the in-series assistance induced a reduction between 38 and 78%. The upper panels in Figure 7 show that parallel RVAD assistance generates a higher increase in total cardiac output compared to in-series assistance at pump speeds higher than 3500 rpm (for each Ees_RIGHT_ value).

**Figure 8:**
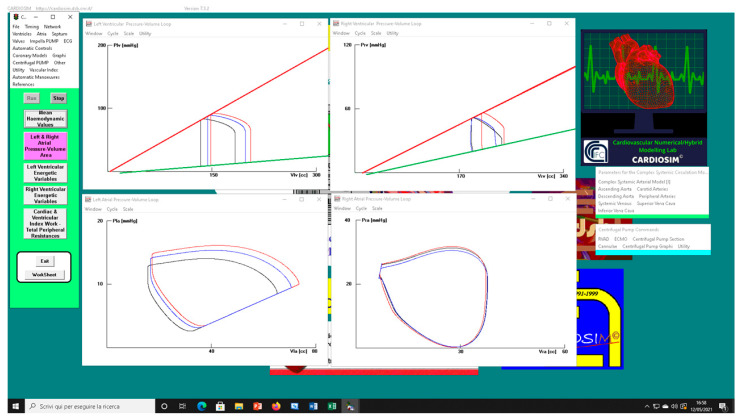
Screen output generated by CARDIOSIM© platform. The left (right) upper panel shows the left (right) ventricular pressure–volume loops when pathological conditions (Ees_RIGHT_ = 0.3 mmHg/mL) (black line), in-parallel RVAD (pump rotational speed at 3000 rpm) (red line), and in-series RVAD assistance (pump rotational speed at 3000 rpm) (blue line) were reproduced by the simulator, respectively. The left (right) lower panel shows the left (right) atrial pressure–volume loops reproduced by CARDIOSIM©.

The left upper (lower) panel in Figure 8 shows the effects induced on the left ventricular (atrial) loop when different types of RVAD assistance were applied to pathological conditions (black loops) reproduced by setting Ees_RIGHT_ to 0.3 mmHg/mL. The assistance was applied in parallel (red loops) and in series (blue loops) mode with a rotational speed of 3000 rpm.

The right upper (lower) panel shows the effects induced on right ventricular (atrial) loops. When in-parallel assistance was applied, a right-sided shift in the left and right ventricular loop (red lines) was observed. Although in-series RVAD assistance did not cause changes in right end-systolic ventricular volume, it led to a reduction in right end-diastolic ventricular loop. The type of assistance did not cause relevant changes in the right atrial loop (right lower panel). 

## 4. Discussion

The left ventricle (LV) was coupled to the low-compliance, high-resistance peripheral arterial circulation and was more adaptable to changes in pressure than volume. In contrast, the right ventricle (RV) was coupled to the high-compliance, low-resistance pulmonary circulation and was more adaptable to changes in volume than pressure. The right ventricle consisted of a free wall containing a wrap-around circumferential muscle at its base and a septum made of oblique helical fibres crossing each other at 60° angles. This was consistent with the helical ventricular myocardial band concept, which defines two interconnected muscle bands: a basal loop with transverse fibres surrounding the left and right ventricles and an apical loop made of a right- and left-handed helix forming an apical vortex [29,30]. The wrap-around transverse fibres constricted or compressed it from leading to a bellows motion responsible for 20% of right ventricular output, whilst the oblique fibres were responsible for shortening and lengthening, which contributed to 80% of right ventricular systolic function [31]. The crista supra-ventricularis shared muscle fibres with the inter-ventricular septum and the free wall played a key anatomical and functional role [32]. A reduction in longitudinal contraction and an increase in transverse shortening were observed following cardiopulmonary bypass and pericardiotomy [33]. This was quite an important aspect to bear in mind and may be addressed initially with pulmonary vasodilators [34]. The relationship between structure and function plays a key role in clinical decision-making, which must be based on detailed knowledge of normality and recognise how a disease can be addressed to restore normality [31]. The important contribution of right ventricular function has been neglected for a long time due to previous observations and assumptions. The onset of right ventricular dysfunction should trigger the search for the main underlying cause in relation to pressure overload, volume overload, or primary myocardial disease [35]. Right heart failure (RHF) is difficult to manage because of its complex geometry and a lack of specific treatments aimed at stabilisation and recovery of right ventricular function. Nevertheless, right ventricular dysfunction remains associated with poor clinical outcome regardless of the underlying disease mechanism [36]. 

A simulation approach overcomes ethical issues and the risk of offering an ineffective or potentially dangerous therapeutic option. At the same time, it may help focus on the specific problem to address. Our starting point was to develop a failing right heart, which would require support at a subsequent stage. The easier way to do it was to act on the ESPVR slope of both ventricles. A range between 0.35 and 0.74 mmHg/mL was observed in patients with pulmonary hypertension [10] with a cut-off of 0.8 for $E_{es}/E_{a}$ ratio as the onset of right ventricular maladaptation. Our initial aim was to observe the effects of RVAD support with either in-series or parallel connection following stepwise variation of right ventricular end-systolic elastance in patients with increased right ventricular afterload. Therefore, the right ventricular end-systolic elastance considered in the present study ranged between 0.3 and 0.8 mmHg/mL as per previously reported values observed in clinical practice [10,37,38]. According to [28], increased native cardiac output was observed in the presence of left ventricular systolic impairment when right ventricular end-systolic elastance increased from 0.1 to 1.0 mmHg/mL. An increased native cardiac output was still observed during VA ECMO support following a stepwise increase in right ventricular end-systolic elastance, but to a lesser degree. A left-to-right ventricular septal shift was observed during diastole following a stepwise increase in right ventricular end-systolic elastance both with and without VA ECMO support. Our considerations were based on the context of pure RVAD support with either in series or parallel connection.

PVA and EW of the left ventricle gain benefit when the pump speed of the RVAD is at least 3500 rpm. The highest effect is obtained when Ees_RIGHT_ is 0.3 mmHg/mL, which is consistent with significant RV dysfunction (left panels in Figure 4). The pathological range considered would suggest that early recognition and aggressive RVAD support is advisable where a lower degree of assistance is required generating enough benefit for a less compromised right ventricle with more potential for recovery. This is an important point to consider in the context of LVAD support when the right heart shows signs of failure requiring attention.

The highest beneficial effect was obtained when the RVAD was connected in parallel to the right ventricle, with up to a nearly 35% increase in total cardiac output (right upper panel in Figure 7) and a lower reduction of right ventricular output compared to in-series RVAD connection. No significant effects were observed on the right atrium regardless of the type of RVAD connection to the right ventricle. Instead, in-parallel RVAD connection had a more beneficial effect on the left atrium. Again, RVAD support has a more beneficial effect on ESV and EDV of the left ventricle when connected in parallel to the right ventricle (left panels in Figure 5). The role of the inter-ventricular septum is critical in this context.

Our aim was to observe the effect of pure RVAD assistance at different stages of right ventricular dysfunction to determine the appropriate timing for intervention. Our target was early graft failure secondary to right heart dysfunction following orthotopic heart transplant and right ventricular failure following LVAD insertion in an apparently preserved right heart function preoperatively. We have focused our attention mainly on Ees_RIGHT_ neglecting Vo. We have not considered the progressive increase in afterload. Despite this limitation, our preliminary findings support the concept of early intervention in the presence of a failing right heart regardless of its aetiology. This simulation study confirms what had been previously advocated, but not always put into practice [39]. A more liberal right ventricular support may be the way forward [40] when considering different support strategies for a failing right ventricle [41]. Late onset of right ventricular failure remains associated with worse survival and higher cumulative incidence of major adverse events [42].

## 5. Conclusions

Despite the limitations of a simulation setting and the limited and not homogeneous availability of haemodynamic data measured in patients during RVAD support, this work allowed a trend analysis of haemodynamic and energetic parameters during pure RVAD support with different connection and at different stages of right ventricular dysfunction. Although RVAD support may be effective in advanced right heart failure, early recognition and aggressive treatment is desirable to achieve a more favourable outcome. RVAD support remains an option for advanced right ventricular failure, although the onset of major adverse events may preclude its use. Our simulation work showed that in-parallel RVAD connection to the right ventricle seems a more suitable option.

## Figures and Tables

**Table 1:** Symbols used in Equations (1) and (2).

Symbol	Description	Unit
*P_lv_(t)* [*P_rv_(t)*]	Instantaneous left (right) ventricular pressure	mmHg
*P_lv,0_* [*P_rv,0_*]	Resting left (right) ventricular pressure	mmHg
*V_lv_(t)* [*V_rv_(t)*]	Instantaneous left (right) ventricular volume	mL
*V_lv,0_* [*V_rv,0_*]	Resting left (right) ventricular volume	mL
*e_lv_(t)* [*e_rv_(t)*]	Left (right) ventricular elastance	mmHg·mL^−1^
*e_Vsp_(t)*	Inter-ventricular septum elastance	mmHg·mL^−1^
*P_la_(t)* [*P_ra_(t)*]	Instantaneous left (right) atrial pressure	mmHg
*P_la,0_* [*P_ra,0_*]	Resting left (right) atrial pressure	mmHg
*V_la_(t)* [*V_ra_(t)*]	Instantaneous left (right) atrial volume	mL
*V_la,0_* [*V_ra,0_*]	Resting left (right) atrial volume	mL
*e_la_(t)* [*e_ra_(t)*]	Left (right) atrial elastance	mmHg·mL^−1^
*e_Asp_(t)*	Inter-atrial septum elastance	mmHg·mL^−1^

**Table 2:** Symbols of the cardiovascular network.

*R_pam_ (R_pas_)*	Main (small) pulmonary arterial resistance [mmHg·cm^−3^·s]
*L_pam_ (L_pas_)*	Main (small) pulmonary arterial inertance [mmHg·cm^−3^·s^2^]
*C_pam_ (C_pas_)*	Main (small) pulmonary arterial compliance [mmHg^−1^·cm^−3^]
*MPAP (SPAP)*	Main (small) pulmonary arterial pressure [mmHg]
*R_par_ (R_pc_)*	Pulmonary arteriole (capillary) resistance [mmHg·cm^−3^·s]
*Wedge*	Pulmonary capillary wedge pressure [mmHg]
*C_vp_*	Pulmonary venous compliance [mmHg^−1^·cm^−3^]
*R_vp_*	Pulmonary venous resistance [mmHg·cm^−3^·s]
*PVP*	Pulmonary venous pressure [mmHg]
*R_ro_ (R_ri_)*	Pulmonary (tricuspid) valve resistance [mmHg·cm^−3^·s]
*SVP*	Systemic veins pressure [mmHg]
*C_VS_*	Systemic veins compliance [mmHg^−1^·cm^−3^]
*R_VS_*	Systemic veins resistance [mmHg·cm^−3^·s]
*R_Vvs_*	Resistor accounting viscous losses of the systemic veins wall [mmHg·cm^−3^·s]
*R_SVC_I_*	First Superior vena cava resistance [mmHg·cm^−3^·s]
*C_SVC_*	Superior vena cava compliance [mmHg^−1^·cm^−3^]
*L_SVC_*	Superior vena cava inertance [mmHg·cm^−3^·s^2^]
*R_SVC_II_*	Second superior vena cava resistance [mmHg·cm^−3^·s]
*SVCP*	Superior vena cava pressure [mmHg]
*LAP(RAP)*	Left (right) atrial pressure [mmHg]
*LVP(RVP)*	Left (right) ventricular pressure [mmHg]
*R_AA_*	Ascending aorta resistance [mmHg·cm^−3^·s]
*R_Vaa_*	Resistor accounting viscous losses of the ascending aorta wall [mmHg·cm^−3^·s]
*L_AA_*	Ascending aorta inertance [mmHg·cm^−3^·s^2^]
*C_AA_*	Ascending aorta compliance [mmHg^−1^·cm^−3^]
*AoP*	Aortic Pressure [mmHg]
*R_DA_*	Descending aorta resistance [mmHg·cm^−3^·s]
*R_Vda_*	Resistor accounting viscous losses of the descending aorta wall [mmHg·cm^−3^·s]
*L_DA_*	Descending aorta inertance [mmHg·cm^−3^·s^2^]
*C_DA_*	Descending aorta compliance [mmHg^−1^·cm^−3^]
*DPA*	Descending aortic pressure [mmHg]
*R_CA_*	Carotid arteries resistance [mmHg·cm^−3^·s]
*R_A_*	Peripheral arteries resistance [mmHg·cm^−3^·s]
*R_Va_*	Resistor accounting viscous losses of the peripheral arteries wall [mmHg·cm^−3^·s]
*C_A_*	Peripheral arteries compliance [mmHg^−1^·cm^−3^]
*PA*	Peripheral arteries pressure [mmHg]
*R_IVC_, R_IVC_I_, R_IVC_II_*	Inferior vena cava resistances [mmHg·cm^−3^·s]
*L_IVC_*	Inferior vena cava inertance [mmHg·cm^−3^·s^2^]
*C_IVC_*	Inferior vena cava compliance [mmHg^−1^·cm^−3^]
*Pt*	Mean intrathoracic pressure [mmHg]
*ela (era)*	Left (right) atrial elastance [mmHg/mL]
*elv (erv)*	Left (right) ventricular elastance [mmHg/mL]
*e_Aspt_ (e_Vspt_)*	Inter-atrial (-ventricular) septal elastance [mmHg/mL]
*Q_artery_ (Q_venous_)*	Coronary arterial (venous) flow [mL/s]
*R_oCANN_ (R_iCANN_)*	RVAD output (input) cannula resistance [mmHg·cm^−3^·s]
*L_oCANN_ (L_iCANN_)*	RVAD output (input) cannula inertance [mmHg·cm^−3^·s^2^]
*C_oCANN_ (C_iCANN_)*	RVAD output (input) cannula compliance [mmHg^−1^·cm^−3^]
*Qli (Qlo)*	Left ventricular input (output) flow [mL/s]
*Qri (Qro)*	Right ventricular input (output) flow [mL/s]
*Qlia (Qria)*	Left (right) atrial input flow [mL/s]

## Data Availability

Not applicable.
